# Erythrocyte osmotic fragility is not linked to vitamin C nutriture in adults with well-controlled type 2 diabetes

**DOI:** 10.3389/fnut.2022.954010

**Published:** 2022-08-12

**Authors:** Ciara Lundy, Samantha N. Fessler, Carol S. Johnston

**Affiliations:** Nutrition Program, College of Health Solutions, Arizona State University, Phoenix, AZ, United States

**Keywords:** vitamin C, diabetes, osmotic fragility, erythrocytes, hypotonic NaCl solutions

## Abstract

Erythrocyte fragility is amplified by oxidative stress and linked to diabetes-specific microvascular disease. Vitamin C supplementation improves glycemic indices in adults with type 2 diabetes (T2D) by improving antioxidant status. This cross-sectional study examined the relationships between vitamin C status and erythrocyte osmotic fragility in adults with or without T2D. Participants provided a fasting blood sample for erythrocyte osmotic fragility testing as a function of hypotonic NaCl concentrations. Additionally, plasma was stabilized with metaphosphoric acid prior to vitamin C analysis using isocratic reverse-phase UV-HPLC separation. Participants were grouped as diagnosed T2D (*n* = 14; 36% female; 55.5 ± 8.2 y; 31.5 ± 9.0 kg/m^2^; HbA1c: 7.4 ± 1.9%; plasma vitamin C: 36.0 ± 12.2 μM) or no diabetes (*n* = 16; 69% female; 38.7 ± 13.5 y; 26.8 ± 6.6 kg/m^2^; HbA1c: 5.4 ± 0.3%; plasma vitamin C: 34.8 ± 10.9 μM). Participant characteristics differed between groups only for age and hemoglobin A1c (HbA1c; *p* < 0.05). All hemolysis parameters were in normal ranges for the participants with T2D, and no significant differences in hemolysis parameters were noted between those with or without T2D. However, among participants with T2D, the NaCl concentration eliciting 50% hemolysis was higher for those with low (<7%) vs. high (>7%) HbA1c values (*p* = 0.037) indicating a slightly higher erythrocyte fragility in the former group. Vitamin C status did not impact any of the hemolysis parameters in adults with or without T2D. Thus, erythrocyte fragility was not elevated in T2D, and vitamin C nutriture was not related to erythrocyte fragility in adults with well-controlled T2D.

## Introduction

Vitamin C deficiency is more prevalent in individuals with diabetes compared to their healthy counterparts (1, 2), a consequence of reduced cellular uptake and recycling of dehydroascorbic acid, and marginal vitamin C status may potentiate the physiological complications associated with the diabetic condition. Hyperglycemia contributes to the overproduction of reactive oxygen species (ROS) by multiple mechanisms, ultimately leading to diabetes-specific microvascular disease, characterized by endothelial dysfunction (3, 4). Specifically, the ROS damage protein moieties in the endothelium including the oxidation of tetrahydrobiopterin (BH4), an essential cofactor for endothelial nitric oxide synthase (eNOS) and the activation of nitric oxide (NO) (4, 5). NO is the key vasodilator released by endothelial cells and responsible for the regulation of vascular tone (4–6). Vitamin C is the premier water-soluble antioxidant *in vivo*, and vitamin C status has been linked to favorable metabolic profiles in patients with type 2 diabetes (7), including improvements in glycemic indices and insulin sensitivity (7–9). Pertinently, at physiological concentrations in normal tissue, vitamin C is linked in a concentration-dependent manner to intracellular BH4 concentrations (10, 11). Vitamin C does not promote the synthesis of BH4 but rather stabilizes the molecule by preventing its oxidation. Hence, there is a demonstrated mechanistic basis for the therapeutic potential of vitamin C for improving endothelial dysfunction in diabetes.

Recently, Tu et al. (12) postulated that diabetes-specific microvascular disease may additionally link to erythrocyte fragility and lysis and that a low vitamin C status would specifically accentuate this pathology. Erythrocytes from individuals with poorly controlled type 2 diabetes (T2D), in comparison to a control sample without T2D, displayed increased mechanical and osmotic fragility (13, 14). In these studies, hemoglobin A1c (HbA1c) concentrations predicted 20%−30% of the variance in erythrocyte fragility. Since HbA1c is a marker of cellular protein glycation, glycosylation status may alter RBC membrane structure causing susceptibility to hemolysis. Samanta et al. demonstrated increased glycosylation in cytoskeletal β-spectrin purified from erythrocytes and suggested this may impair erythrocyte membrane integrity and sensitivity to hemolysis (15). Erythrocyte osmotic fragility is noted in Gulo^−/−^ knockout mice unable to synthesize vitamin C and attributed to the decreased production of cytoskeletal β-spectrin (12). Sanford et al. (16) demonstrated that the decrease in β-spectrin levels in erythrocytes during storage due to oxidative modifications was reversed by the addition of vitamin C to the samples. These data demonstrate detrimental effects of oxidative stress on erythrocyte fragility and further support the importance of maintaining adequate vitamin C nutriture in individuals with diabetes.

Although many studies have profiled the metabolic changes related to oxidative stress and inflammation in individuals with diabetes as a function of vitamin C status, only one trial has specifically examined erythrocyte fragility in T2D in relation to vitamin C nutriture (12). This study examined the relationships between vitamin C status and erythrocyte osmotic fragility in adults with or without T2D.

## Materials and methods

### Participants and experimental design

Adults aged 18–65 y were recruited from a large campus community and neighboring localities in Phoenix, Arizona during the late spring and summer of 2021 using listservs, posted flyers, campus advertisements, and social media. Both healthy individuals without T2D (ND) and individuals diagnosed with T2D for at least 1 year were screened for smoking, pregnancy, vegetarianism, type 1 diabetes, active or unresolved health complications including cardiovascular disease, chronic kidney disease, cancer, respiratory diseases, and/or recent injury or surgery. Those with a history of vitamin C supplementation (>60 mg/day within the previous 3 months) were also excluded from study participation. All participants provided written consent, and the study was approved by the Arizona State University Institutional Review Board (STUDY00013197). COVID-19 precautions were followed at study appointments including masking, temperature checks, and COVID symptoms clearance.

A cross-sectional study design was employed, and participants met with investigators on one occasion. Participants reported to the test site in a rested, fasted state (no food or beverage for 10 h except for water). Height was measured using a stadiometer and body composition was measured using a calibrated bioelectrical impedance scale (Cat. No. TBF-300, Tanita, Arlington Heights, IL). Participants completed a health history questionnaire and a survey on fruit and vegetable intake, and provided a venous blood sample.

### Primary outcome measurements

Whole blood was collected into vacutainers containing K_2_EDTA preservative. A sample was sent to Sonora Quest Laboratories (Phoenix, AZ) for hematocrit and HbA1c measurement. Whole blood for the erythrocyte fragility analysis was processed as outlined by Parpart et al. (17). For each blood sample, aqueous NaCl solutions (2 ml, in duplicate) for 0.90, 0.70, 0.65, 0.60, 0.55, 0.50, 0.45, 0.40, 0.35, 0.30, 0.20, and 0% NaCl (w/v) were prepared using distilled water. A 20 μl aliquot of whole blood was added to each solution, and the tubes were slowly inverted several times to gently mix and incubated at 20°C for 30 min. Samples were centrifuged for 10 min (20,00 g at 4 °C), and absorbance of the supernatant was measured at 540 nm (Thermo Fisher Genesys UV/VIS spectrophotometer). Hemolysis in each tube was expressed as a percentage, taking as 100% the maximum value of absorbance at 0%. The percent of hemolysis was calculated according to the equation: % Hemolysis = (O.D. of test well - O.D. of 0.90% NaCl well) ÷ (O.D. of dH2O well - O.D. of 0.90% NaCl well). H50 was the extrapolated concentration of NaCl that caused 50% of erythrocyte hemolysis.

For vitamin C analyses, blood samples were rapidly centrifuged (8 min; 2,800 g at 4°C), and the supernatant was mixed with an equal volume of 10% (w/v) metaphosphoric acid (MPA) in 2 mmol/L disodium EDTA. The mixture was placed in ice for 15 min prior to centrifuging (10 min; 4,700 g at 4°C), and the supernatant was aliquoted into micro-centrifuge tubes for storage at −80°C until analyzed. Ascorbic acid was measured by reverse-phase HPLC-UV analysis (18). Equal volumes of 5 mmol/L Tris (2-carboxyethyl) phosphine (TCEP) and HPLC grade water were added to sample tubes and kept in the dark for 20 min at room temperature to react. TCEP was used as a reducing agent allowing for measurement of the total vitamin C content of the sample. Samples were then centrifuged at 16,000 g for 5 min. Centrifuge tubes were kept on ice and the supernatant was transferred into HPLC vials for analysis. Samples were analyzed immediately or kept in the refrigerated auto-sampler for up to 4 h. Analysis was carried out using an HPLC (Waters Alliance e2695) equipped with a photodiode array detector and a column and guard (Agilent Zorbax Eclipse XDB-C18). The column was held at 25°C and the sample volume was set to 20 μL. Separation was achieved using an isocratic 1.8 mmol/L sulfuric acid mobile phase with a flow rate of 0.8 ml/min.

### Statistical analysis

Statistical analysis was performed using the IBM SPSS statistical software (IBM SPSS Statistics for Windows, Version 25.0. Armonk, NY: IBM Corp.). Data are reported as mean ± standard deviation (SD) for descriptive statistics, hemolysis, and blood vitamin C concentrations for study groups. Data were tested for normality, and univariate analysis and multivariate analysis of variance were used to determine differences between groups controlling for age and BMI. Spearman's rho was used to assess relationships between variables. The significance level was set at *p* ≤ 0.05.

## Results

### General characteristics of the participants

Participants (14 males, 16 females) ranged from 20 to 64 years of age (46.5 ± 14.0 y), and the participants with T2D (*n* = 14) were older on average in comparison to participants without T2D (Table 1). However, age was not correlated to erythrocyte hemolysis or to plasma vitamin C concentrations (*r* = −0.026, *p* = 0.891 and *r* = 0.087, *p* = 0.652, respectively). Participants were overweight (29.0 ± 8.1 kg/m^2^), but the frequency of obesity did not vary between participants with or without T2D (23 and 13%, respectively, *p* = 0.299). Daily fruit and vegetable intake did not differ between groups (2.5 ± 1.3 and 2.9 ± 1.3 servings for participants with and without T2D, *p* = 0.820), and intakes mirrored the average intake reported for American adults, 2.7 servings/day (19). HbA1c ranged from 4.8 to 11.1% (mean, 7.4 ± 1.9%) and 4.8 to 6.0% (mean, 5.4 ± 0.3%) in the participants with and without T2D, respectively (Table 1). Plasma vitamin C concentrations ranged from 19 to 63 μM among participants, and marginal vitamin C status (<28 μM; ref. (20)) was noted for 29% of participants with T2D and 25% of participants without T2D (*p* = 0.825). The mean plasma concentrations did not differ between participant groups (36.0 ± 12.2 and 34.8 ± 10.9 μmol for participants with and without T2D, respectively; Table 1).

**Table 1:** Comparison of characteristics and biochemical parameters for participant groups.

	**Reference**	**Groups, mean** ±**SD**				***p*** **-value**	
		**ND**	**T2D**	**T2D (HbA1c <7.0%)**	**T2D (HbA1c >7.0%)**	**ND vs. T2D**	**T2D HbA1c <7 vs. >7%**
Gender (M/F)		5/11	9/5	3/4	6/1	0.149	
Age (year)		38.7 ± 13.5	55.5 ± 8.2	57.4 ± 7.9	53.6 ± 8.8	<0.001	0.402
Fruit/vegetable intake (serving/day)	5	2.9 ± 1.3	2.5 ± 1.3	3.0 ± 1.6	2.0 ± 0.6	0.820	0.364
Body mass index (kg/m^2^)	18.5–24.9	26.8 ± 6.6	31.5 ± 9.0	28.1 ± 8.2	35.0 ± 9.0	0.325	0.240
Hematocrit (%)	M:38.3–48.6 F:35.5–44.9	44.4 ± 3.2	45.8 ± 5.7	43.9 ± 5.6	47.6 ± 5.5	0.518	0.224
Plasma vitamin C (μM)	>28	34.8 ± 10.9	36.0 ± 12.2	32.2 ± 9.7	39.8 ± 14.0	0.369	0.677
HbA1c (%)	3.0–5.9	5.4 ± 0.3	7.4 ± 1.9	5.9 ± 0.6	8.8 ± 1.5	0.009	0.001
H50 (%NaCl w/v)	0.40–0.45	0.433 ± 0.016	0.431 ± 0.018	0.436 ± 0.021	0.425 ± 0.015	0.431	0.037

*Values are presented as means standard deviation*.

*ND, no diabetes (n = 16); T2D, type 2 diabetes (n = 14); H50, the extrapolated concentration of NaCl that caused 50% of erythrocyte hemolysis*.

*p-value represents univariate analyses controlling for age and BMI*.

### Primary outcomes

**Figure 1:**
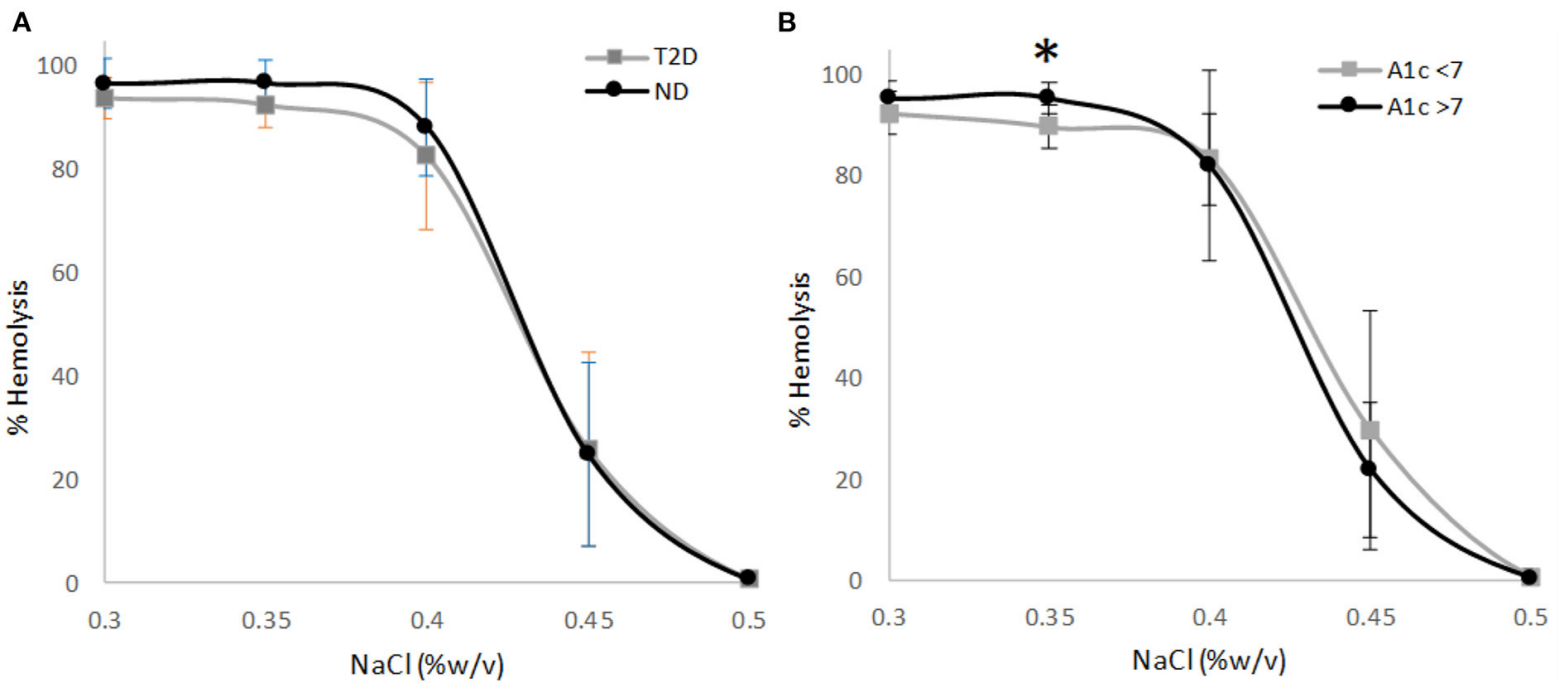
**(A)** Osmotic erythrocyte fragility in participants with type 2 diabetes (T2D; *n* = 14) or normal glucose tolerance (ND; *n* = 16) as a function of NaCl concentration (*p* = 0.090). **(B)** Osmotic erythrocyte fragility in the T2D participants grouped by hemoglobin A1c status as a function of NaCl concentration (*n* = 7/group; *p* = 0.031). Data points represent mean ± SD; *p*-values represent multivariate analysis controlling for age and BMI. Asterisk denotes a significant difference between groups (*p* < 0.05).

The osmotic fragility of erythrocytes as a function of hypotonic salt concentrations were within the reference ranges for all but one ND participant. Erythrocyte hemolysis was initiated at 0.45% NaCl for all study participants (reference range: 0.45%−0.50% NaCl; ref. (14)). The 50% erythrocyte hemolysis inflection point ranged from 0.40 to 0.45 for all study participants (reference range: 0.40%−0.45% NaCl; ref. (14)), and the 100% erythrocyte hemolysis inflection point was <0.35% (reference: <0.34%; ref. (14)) for all study participants except for one ND participant with the inflection point at 0.40% NaCl. The osmotic fragility curves for erythrocytes as a function of NaCl concentration did not vary between comparison groups until erythrocyte hemolysis was nearly complete at 0.35% NaCl. Participants without T2D had a modestly higher erythrocyte hemolysis rate at 0.35% NaCl in comparison to participants with T2D (+4%; *p* = 0.090 for multivariate analysis; Figure 1A). In the participants with T2D, those with higher HbA1c values (>7%) displayed a significantly higher rate of hemolysis at 0.35% NaCl (+6%; *p* = 0.031 for multivariate analysis; Figure 1B) in comparison to their counterparts with HbA1c values <7%, indicating a greater degree of erythrocyte fragility in high HbA1c group. H50 did not differ between participants with or without T2D. However, among participants with T2D the NaCl concentration eliciting 50% hemolysis was significantly higher for participants with low HbA1c concentrations (<7%) in comparison to their counterparts with higher HbA1c values indicating greater erythrocyte stability in the latter group (Table 1). HbA1c was not significantly correlated to the NaCl concentration eliciting 50% erythrocyte hemolysis in participants with T2D (*r* = −0.330, *p* = 0.249).

**Figure 2:**
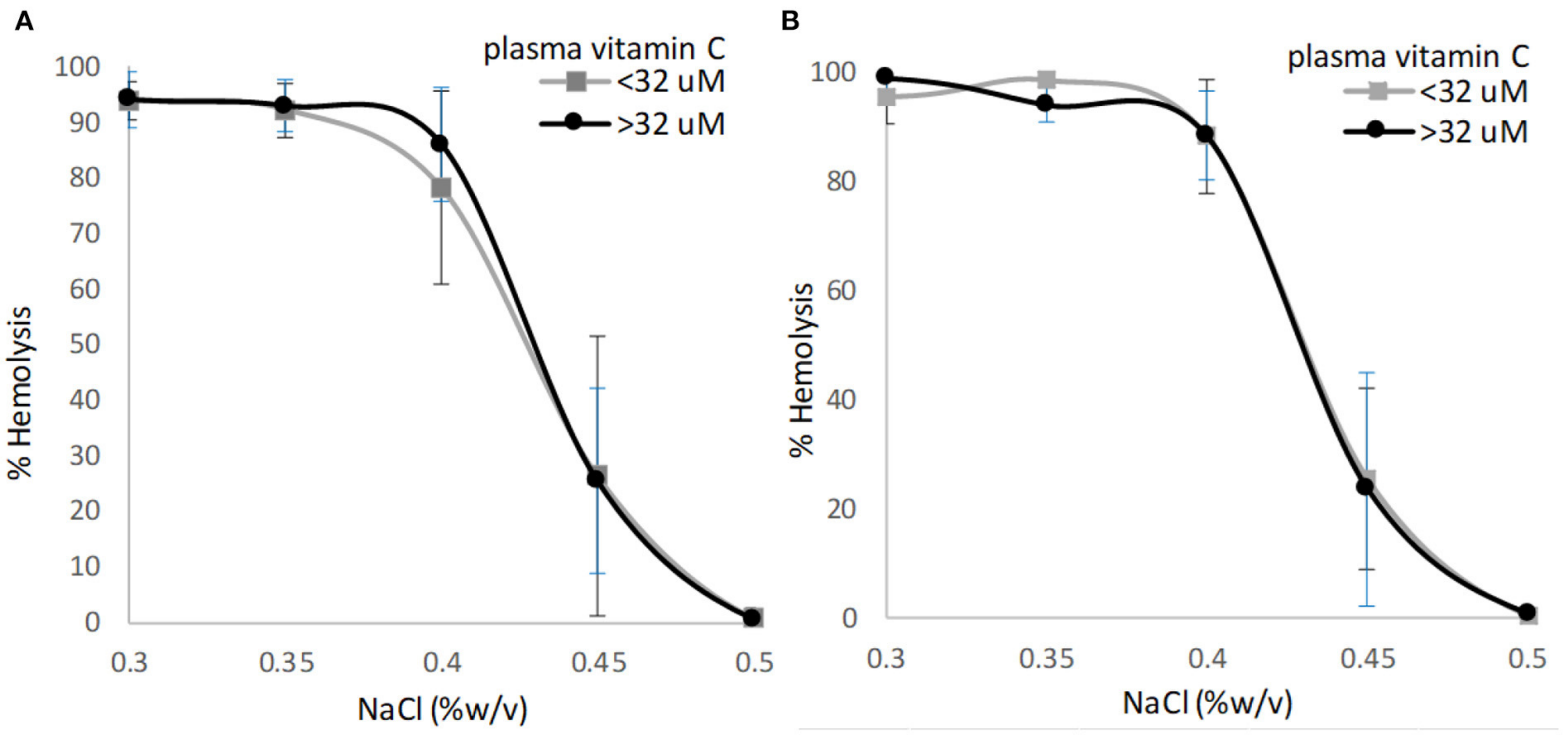
Osmotic erythrocyte fragility as a function of plasma vitamin C nutriture using the sample median (32 μM) as the cutoff. **(A)** Individuals with type 2 diabetes, *p* = 0.532; **(B)** Individuals with no diabetes, *p* = 0.170. Data points are mean ± SD; *p*-value represents multivariate analysis controlling for age and BMI.

Plasma vitamin C concentrations were not correlated to the NaCl concentration eliciting 50% erythrocyte hemolysis for participants with T2D (*r* = −0.042, *p* = 0.887) or for participants without T2D (*r* = 0.240, *p* = 0.390). The osmotic fragility curves for erythrocytes as a function of NaCl concentration and plasma vitamin C status did not differ for participants with or without T2D (Figures 2A,B; *p* = 0.532 and *p* = 0.170 for multivariate analysis, respectively). The extrapolated NaCl concentration eliciting 50% erythrocyte hemolysis did not differ significantly among participants with or without T2D when grouped by high (>32 μM) vs. low (<32 μM) plasma vitamin C concentrations (Table 2).

**Table 2:** Comparison of biochemical parameters for participant groups as a function of vitamin C status.

**Group**	**Plasma vitamin C**		***p-*Value**
	**<32 μM**	**>32 μM**	
T2D	*n* = 5	*n* = 9	
Plasma vitamin C (μM)	24.3 ± 4.3	42.5 ± 10.1	0.006
H50 (%NaCl w/v)	0.434 ± 0.028	0.429 ± 0.012	0.321
ND	*n* = 8	*n* = 7	
Plasma vitamin C (μM)	27.4 ± 3.6	43.4 ± 10.3	0.044
H50 (%NaCl w/v)	0.430 ± 0.013	0.436 ± 0.019	0.904

*ND, no diabetes (n = 16); T2D, type 2 diabetes (n = 14); H50, the extrapolated concentration of NaCl that caused 50% of erythrocyte hemolysis. p-value represents univariate analyses controlling for age and BMI*.

## Discussion

These data suggest that erythrocyte fragility is not necessarily elevated in individuals with well-controlled T2D, and furthermore, plasma vitamin C concentrations were not correlated with erythrocyte fragility in the sample as a whole or in participants with or without T2D. The sharply sigmoidal hemolysis curves generated in this study as a function of increasing hypotonic salt concentrations closely mirror reference curves based on the inflection points for the initiation of erythrocyte hemolysis and for 50 and 100% erythrocyte hemolysis (14). A broadening or more ‘linear' hemolysis curve (e.g., initiation of hemolysis at a higher salt concentration), which would indicate increased cell fragility (21), were not noted in this study. The evidence to date is mixed regarding erythrocyte fragility in diabetes. Kung et al. (14) reported increased erythrocyte fragility in Chinese adults with T2D for at least 5 years (*n* = 45; mean HbA1c = 8.5%). However, Rownak et al. (22) did not observe increased erythrocyte fragility in 100 newly diagnosed T2D from Bangladeshi (mean HbA1c = 9.1%) in comparison to a matched control group without T2D. Ibanga et al. (23) did note a significant increase in erythrocyte fragility in Nigerian adults with T2D (*n* = 75; mean HbA1c = 8.2%) in comparison to controls, but erythrocyte fragility was not related to duration of diabetes. In an Indian population, Adeshara et al. (24) noted a 1.6-fold increase in osmotic fragility in erythrocytes from Indian adults with T2D (*n* = 85; mean HbA1c ~10%) compared to a control group without T2D. However, the mean H50 values in this patient sample was markedly elevated (~0.75 %NaCl), as was the mean for the control group (~0.48 %NaCl), suggesting an underlying factor influencing erythrocyte fragility in this previous report (24).

Several investigations reporting increased erythrocyte fragility among individuals with T2D linked erythrocyte fragility directly to HbA1c values (14, 24), yet other trials did not observe a significant relationship between these parameters (22, 23). The T2D group in the present report would be considered “well controlled” based on the mean HbA1c value 7.4% (desired target range: 7%−8% (25)), which may account in part to the lack of difference in erythrocyte osmotic fragility between those with or without T2D. Among the participants with T2D, erythrocyte fragility was slightly improved at H50 for those with HbA1c >7%; however, when nearing 100% hemolysis (hypotonic salt concentration at 0.35%) the erythrocytes from the elevated HbA1c group demonstrated a 6% higher degree of hemolysis when compared to participants in the low HbA1c group. These deviations are small in magnitude and all hemolytic values for the participants with T2D fell within the normal ranges expected for *ex vivo* hemolysis of erythrocytes by osmotic pressure. In the earlier reports, patient groups averaged higher mean HbA1c values (8.2%−9.1%), and the mean H50 values fell above the 0.40%−0.45% NaCl reference range, clearly suggestive of increased erythrocyte fragility.

The reported increased osmotic fragility of erythrocytes from patients with T2D has been attributed to red cell membrane changes leading to increased rigidity (e.g., reduced deformability), a result of high blood glucose concentrations, high glycosylation rates, and increased oxidative stress (26). In a series of *ex vivo* investigations, Nigra et al. (27) demonstrated that under conditions of high physiologic glucose concentrations, erythrocyte cytosolic tubulin is acetylated at higher rates and relocated to the cell membrane. The increase in membrane acetylated tubulin altered membrane properties, which correlated with a decrease in membrane deformability. The acetylation of tubulin was quickly reversed when glucose concentrations decreased. Thus, individuals with T2D that is well controlled (as in the present report) may not be prone to the erythrocyte changes linked to reduced deformability and increased fragility.

This report examined the relationship between erythrocyte fragility and plasma vitamin C concentrations in individuals with or without T2D. The H50 values and osmotic fragility curves for erythrocytes as a function of plasma vitamin C status (>32 vs. <32 μM) did not differ for participants with T2D. Moreover, the H50 values did not differ significantly by vitamin C status, and the sigmoidal hemolysis curves for all groups closely mirrored reference curves. There is one previous report examining the relationship between erythrocyte fragility and vitamin C status in individuals with and without T2D (total *n* = 39); however, the H50 values in this report are below reference ranges for both population groups (0.32%−0.38% NaCl) suggesting a high resistance to erythrocyte lysis regardless of vitamin C status (12). In this same report, in Gulo-/- knockout mice unable to synthesize vitamin C, erythrocyte fragility was significantly improved with vitamin C supplementation as indicated by a 13% reduction in H50 (0.45% NaCl for mice not supplemented and 0.39% NaCl for supplemented mice) (12).

Participants in the present study had low plasma vitamin C concentrations, a reflection of the screening protocol that excluded individuals that supplemented vitamin C. These data mirror national survey data for adults who do not supplement vitamin C (plasma vitamin C range: 32–40 μM; ref. (28)). The data herein demonstrated that in healthy adults and in adults with well controlled T2D, erythrocyte fragility data fall within reference values even when plasma vitamin C concentrations averaged in the low-marginal range (<32 μM; see Table 2).

### Limitations

These data represent only the second investigation of the relationship between vitamin C status, the diabetic state, and erythrocyte fragility. The small sample sizes and the cross-sectional study design limit data interpretation. Furthermore, based on HbA1c concentrations, the participants with T2D were on average well controlled, and their erythrocyte fragility was not elevated above reference values. Future investigations in a less well-controlled patient sample displaying increased erythrocyte fragility are needed to determine if vitamin C supplementation improves erythrocyte fragility in diabetes.

Although these data did not link vitamin C status to erythrocyte fragility in patients with well controlled diabetes, there are indications that vitamin C supplementation can improve other conditions associated with T2D. A 2021 meta-analysis encompassing 28 studies and 1,574 participants concluded that vitamin C supplementation (500–1,000 mg/day) was beneficial for improving glycemic control and reducing cardiovascular disease risk factors in T2D (29). Clinically significant reductions in HbA1c (−0.54%) and systolic and diastolic blood pressures (−6.3 and −3.8 mmHg, respectively) were achieved with vitamin C supplementation. In this analysis, vitamin C supplementation also lowered malondialdehyde suggesting a reduction in oxidative stress. These findings remained statistically significant and clinically relevant when only “low-risk-of-bias” studies were examined (29). In a separate meta-analysis comprising 13 trials, Ashor et al. (30) demonstrated that vitamin C supplementation (median dosage, 1,000 mg/day; median duration 30 days) significantly improved fasting blood glucose concentrations in individuals with T2D (−0.44 mmol/l, 95% CI: −0.81, −0.07, *p* = 0.02). Others have linked the improvements in glycemic indices by vitamin C supplementation to improvements in the antioxidant status of patients (7–9). Vitamin C is also linked to improved endothelial function in patients with T2D (10, 11) reducing risk for cardiovascular disease.

## Conclusion

These data did not link marginal vitamin C status to increased erythrocyte fragility in adults with or without T2D. Moreover, an increased erythrocyte fragility was not observed for the participants with T2D when compared to the participants without T2D. However, to fully understand whether vitamin C nutriture contributes to erythrocyte fragility and endothelial dysfunction in diabetes, future research should examine the impact of vitamin C supplementation on erythrocyte fragility in individuals with T2D and HbA1c concentrations above 8%.

## Data availability statement

The raw data supporting the conclusions of this article will be made available by the authors, without undue reservation.

## Ethics statement

The studies involving human participants were reviewed and approved by Institutional Review Board at Arizona State University. The patients/participants provided their written informed consent to participate in this study.

## Author contributions

CL and CJ conceptualized the study, contributed to formal analysis, writing the original draft, and funding acquisition. All authors contributed to methodology, investigation, supervision, writing, reviewing, editing the manuscript, and approved the submitted version.

## Conflict of interest

The authors declare that the research was conducted in the absence of any commercial or financial relationships that could be construed as a potential conflict of interest.

## Publisher's note

All claims expressed in this article are solely those of the authors and do not necessarily represent those of their affiliated organizations, or those of the publisher, the editors and the reviewers. Any product that may be evaluated in this article, or claim that may be made by its manufacturer, is not guaranteed or endorsed by the publisher.
